# Gelatin Nanoemulsion-Based Co-Delivery of Terbinafine and Essential Oils for Treatment of *Candida albicans* Biofilms

**DOI:** 10.3390/microorganisms13010127

**Published:** 2025-01-09

**Authors:** Muhammad Aamir Hassan, Sadaf Noor, Jungmi Park, Ahmed Nabawy, Maitri Dedhiya, Robin Patel, Vincent M. Rotello

**Affiliations:** 1 Department of Chemistry, University of Massachusetts Amherst, 710 North Pleasant Street, Amherst, MA 01003, USA; muhammadaami@umass.edu (M.A.H.); snoor@umass.edu (S.N.); jungmipark@umass.edu (J.P.); anabawy@umass.edu (A.N.); mdedhiya@umass.edu (M.D.); 2 Institute of Molecular Biology and Biotechnology, Bahauddin Zakariya University, Multan 60800, Pakistan; 3 Division of Clinical Microbiology, Department of Laboratory Medicine and Pathology, Mayo Clinic, 200 First Street SW, Rochester, MN 55905, USA; patel.robin@mayo.edu

**Keywords:** fungal infection, biofilm, combination therapy, gelatin, nanoemulsions, synergy

## Abstract

Fungal infections represent a significant global health challenge. *Candida albicans* is a particularly widespread pathogen, with both molecular and biofilm-based mechanisms making it resistant to or tolerant of available antifungal drugs. This study reports a combination therapy, active against *C. albicans*, utilizing terbinafine and essential oils incorporated into a gelatin-based nanoemulsion system (T-GNE). Eugenol and methyl eugenol/terbinafine T-GNEs had an additive efficacy, while carvacrol (CT-GNE) worked synergistically with terbinafine, providing effective antifungal treatment with minimal mammalian cell toxicity. Confocal microscopy demonstrated that CT-GNE penetrated the dense *C. albicans* biofilm and disrupted the fungal cell membrane. Overall, the combination of essential oils with terbinafine in GNE provided a promising treatment for fungal biofilms.

## 1. Introduction

Fungal infections are a growing concern globally, resulting in over 150 million severe cases and 2 million deaths annually worldwide [1]. The *Candida albicans* is a major contributor to invasive candidiasis and has significant mortality in immunocompromised patients [2]. Approximately 75% of candidal infections are caused by *C. albicans*, amounting to societal costs of an estimated $2 billion to the global healthcare system annually [3,4].

Antifungal drugs, including terbinafine, are generally active against planktonic fungal cells but less so against mature biofilms [5]. In addition, fungi may harbor acquired resistance; *C. albicans,* for example, has acquired the resistance to multiple antifungal drugs through mechanisms including genetic adaptations, the modulation of permeability, the overexpression of drug targets, and active efflux pumps to reduce drug accumulation [6,7,8]. The *C. albicans* forms dense biofilms, making treatment more challenging by creating a physical barrier of extracellular polymeric substances (EPS) alongside their physiological tolerance [9]. The EPS matrix of biofilms can shield fungal cells from the host immune responses and conventional antifungal drugs [10]. Importantly, fungal biofilms are more anatomically complex than bacterial biofilms because eukaryotes can assume various morphologies within the biofilm, including forming multicellular hyphae and unicellular yeast [11].

Phytochemicals such as essential oils, which are generally recognized as safe (GRAS), are promising alternative antifungal agents [12]. They have high biocompatibility, broad-spectrum antimicrobial potential, and a low risk of resistance development due to their multiple modes of action [13]. However, essential oils are highly hydrophobic and poorly active against fungal biofilms because of their insolubility in aqueous media and restricted entry to highly negatively charged biofilm EPS matrices [14].

Nanocarrier systems of surfactant polymers and nanoparticles provide a strategy for treating fungal biofilms [15,16,17,18]. Natural polymers such as gelatin, which is GRAS, provide promising nanocarrier systems to deliver hydrophobic therapeutic drugs and essential oils [19,20]. Gelatin has hydrophilic and hydrophobic constituents that enable the loading of hydrophobic drugs to increase their solubility [21,22]. Biopolymeric nanocarriers such as gelatin have been shown to enhance the overall activity of essential oils by improving their solubility, biofilm penetration, and targeting of fungal cells [23,24,25]. This strategy offers the potential for delivering loaded therapeutic cargo across biofilm matrices and combating fungal biofilm-associated infections [26,27].

Combination therapy is a strategy to enhance antifungal efficacy [28]. Combining essential oils with conventional antifungal drugs may enhance the therapeutic efficacy for the treatment of fungal biofilms while minimizing off-target effects [29]. This approach can also help overcome selection of resistance, which occurs with conventional drugs that have single targets [30,31].

The incorporation of antifungal drugs, such as terbinafine, into nanocarrier systems could improve the penetration of antifungal agents into biofilms, increasing their utility. In this study, terbinafine and essential oil-loaded gelatin nanoemulsion (T-GNE) scaffolds were fabricated and had their efficacy demonstrated against *C. albicans* IDRL-7034 biofilms. Terbinafine was suspended into essential oils (i.e., carvacrol, eugenol, methyl eugenol) and loaded into gelatin nanoemulsions (GNEs). A checkerboard analysis based on minimal biofilm inhibitory concentration (MBIC) demonstrated synergistic effects with carvacrol and terbinafine in gelatin nanoemulsions (CT-GNEs) and additive effects with eugenol and methyl eugenol (ET-GNE and MT-GNE, respectively). The antibiofilm activity of T-GNEs was evaluated against 2-day biofilms of *C. albicans,* showing a 4-fold enhancement of CT-GNE and 2-fold of ET-GNE and MT-GNE activity. CT-GNE penetrated the dense biofilms and reduced resident fungal cells while exhibiting minimal toxicity to mammalian cells. Significantly, *C. albicans* did not develop resistance to T-GNEs even after their repeated exposure to sub-inhibitory concentrations, unlike terbinafine alone, which exhibited a 9-fold rise in MIC after just five passages. The synergistic efficacy of CT-GNEs provides a promising platform for treating biofilm-associated fungal infections.

## 2. Materials and Methods

All reagents and solvents were purchased from Thermo Fisher Scientific and used as received. *C. albicans* IDRL-7034 (Mayo Clinic in Rochester, MN, USA), isolated from a pelvic abscess, was studied. For the preparation of overnight cultures of *C. albicans*, a single colony from agar plates was grown in tryptic soy broth (TSB) supplemented with 1% glucose at 30 °C with 220 rpm agitation. Human fibroblast NIH/3T3 cells (ATCC CRL-1658) were purchased from the American Type Culture Collection (ATCC, Manassas, VA, USA). For cell culture, Dulbecco’s Modified Eagle’s Medium (DMEM; ATCC 30-2002) and fetal bovine serum (SH3007103) from Thermo Fisher Scientific (Waltham, MA, USA) were used. The Invitrogen™ AlamarBlue™ Cell Viability Reagent (DAL 1100) from Thermo Fisher Scientific was used following the manufacturer’s instructions.

### 2.1. Fabrication and Characterization of Terbinafine-Loaded Gelatin Nanoemulsions

GNEs were prepared by emulsifying essential oils, including carvacrol, eugenol, and methyl eugenol, into a gelatin stock solution. Specifically, 3 µL of each oil were mixed with 497 µL of gelatin stock solution (240 µg/mL) and emulsified for 50 s using an amalgamator (Zoneray HL-AH G8). For oil–terbinafine-loaded gelatin nanoemulsions (T-GNEs), 133 mg of terbinafine was suspended into 1 mL of carvacrol, eugenol, or methyl eugenol. Then, 3 µL of this mixture was used in 497 µL of gelatin solution to fabricate the T-GNEs (100% = 6 mg/mL of oil with ~800 µg/mL of terbinafine). Dynamic light scattering (DLS) was used to identify the size of T-GNEs, and the zeta potential was used for the charge estimation.

### 2.2. Assessment of Minimal Inhibitory Concentrations (MICs)

The mid-log phase fungal culture was grown in 4–5 mL TSB with 1% glucose from the 100 µL overnight culture for 6–8 h at 30 °C with 220 rpm agitation. Mid-log phase cultures were harvested by brief spinning at 4000 rpm for 5 min. Fungal cells were resuspended in 0.85% NaCl solution and washed by centrifugation 3 times. The optical density (OD) was assessed at 600 nm. The fungal culture solution was diluted using an M9 + TSB with 1% glucose (9:1) medium to make a solution of 1 × 10⁶ CFU/mL. The 2-fold dilution of GNEs, T-GNEs, and terbinafine was prepared in 50 µL of M9 + TSB with 1% glucose. A total of 50 µL of 1 × 10⁶ CFU/mL fungal culture were mixed with the testing material in 96-well microtiter plates, including media sterile (negative) and fungal culture (positive) control. This plate was incubated overnight at 220 rpm at 30 °C. The MIC values were analyzed with a plate reader through OD_600._

### 2.3. Minimal Biofilm Inhibitory Concentration (MBIC) Analysis

The fungal mid-log phase cultures were collected as mentioned above. The fungal culture solution was diluted using an M9 + TSB with 1% glucose (9:1) medium to make 1 × 10^8^ CFU/mL solution. A total of 150 µL of 1 × 10^8^ CFU/mL fungal culture was added to a 96-well microtiter plate with a pegged lid and incubated at 30 °C for 6 h at 50 rpm, after which the pegged lid was rinsed with 200 µL PBS and submerged into a 96-well plate with two-fold diluted T-GNEs and terbinafine for 24 h at 30 °C. The MBIC values were defined as the minimal concentration of T-GNEs and terbinafine which inhibited the biofilm formation on the pegged lids, which was assessed by OD_600_ with a plate reader after incubation.

### 2.4. Evaluation of Fractional Inhibitory Concentration (FIC)

A 2D checkerboard titration assay was used, as previously reported [25], to assess the interaction between the essential oils, carvacrol, eugenol, and methyl eugenol, with terbinafine in GNEs through MBIC. Two-fold dilutions of each GNE, as well as terbinafine, were prepared in M9 + TSB 1% glucose media according to their MBICs against *C. albicans* IDRL 7034 and incubated overnight at 30 °C. The OD_600_ was taken to evaluate the MBICs of each material alone and in combination. The FICs indices were calculated using the following formulae.

FIC_Terbinafine_ = MBIC_T-GNE_ ÷ MBIC_Terbinafine_

FIC_T-GNE_ = MBIC_T-GNE_ ÷ MBIC_GNE_

FIC index = FIC_Terbinafine_ + FIC_T-GNE_

### 2.5. Antibiofilm Activity Assessment of T-GNE

A fungal culture of 0.1 OD_600_ was prepared in M9 + TSB supplemented with 1% glucose and 100 µL were seeded to each well of a 96-well microtiter plate for biofilm cultivation with negative media controls. The plate was incubated for 48 h at room temperature in static conditions. Before treatment, the plate was washed with PBS to remove the planktonic cells and treated with 100 µL of various concentrations of T-GNEs for 3 h at 37 °C. After the treatment, the liquid was removed from each well and the wells were washed with PBS. The fungal cell viability was assessed using an AlamarBlue assay. A total of 110 µL of 1 × AlamarBlue reagent was added to each well, followed by an incubation of 2 h at 37 °C. The fluorescence readings at excitation/emission, 560 nm/590 nm, were taken, and the fungal cell viability (%) was calculated. The experiment was repeated in triplicate on two separate occasions.

### 2.6. Biofilm Penetration Study

Two-day-old *C. albicans* IDRL-7034 biofilms were grown on confocal dishes using the abovementioned procedures. A treatment of 2 h was applied with the 8% CT-GNEs, followed by washing with PBS. Propidium iodide (PI) and SYTO 9 dyes were used for live/dead staining. The staining solution was prepared with 1.65 μM PI and 10 μM SYTO 9, and samples were stained for 1 h. The biofilm was rinsed and observed using confocal laser scanning microscopy (CLSM) with a Nikon A1 resonant scanning confocal and TIRF module. The images were processed using NIS-Elements software (5.21.00, Build 1483).

### 2.7. Fibroblast Cells Viability with T-GNEs

Human fibroblast NIH/3T3 cells were seeded into 96-well microtiter plates at a density of 20,000 cells per well in DMEM supplemented with 10% fetal bovine serum and 1% penicillin–streptomycin. The plates were incubated at 37 °C in a humidified atmosphere with 5% CO_2_ overnight. Before treatment, the seeded cells were washed with PBS and treated with the T-GNEs for 3 h. The mammalian cell viability was assessed using an AlamarBlue assay by taking the fluorescence at excitation/emission: 560 nm/590 nm. The experiment was repeated twice, separately, to record the results in triplicates

### 2.8. Resistance Response to Sub-Inhibitory Concentration

The *C. albicans* was cultured in TSB with 1% glucose along with a sub-lethal dose (one-third of the MIC) of terbinafine and T-GNEs overnight at 37 °C at 220 rpm. The next day, the fungal cells grown at these concentrations were harvested, streaked on agar, and used to evaluate the MICs of T-GNEs and terbinafine against these cells, respectively. Each sequential passage was prepared by treating fungal cells to one-third of the MBIC of the previous therapeutic dosage by up to 5 passages.

## 3. Results

### 3.1. Fabrication and Characterization of Terbinafine-Loaded Gelatin Nanoemulsions

**Figure 1:**
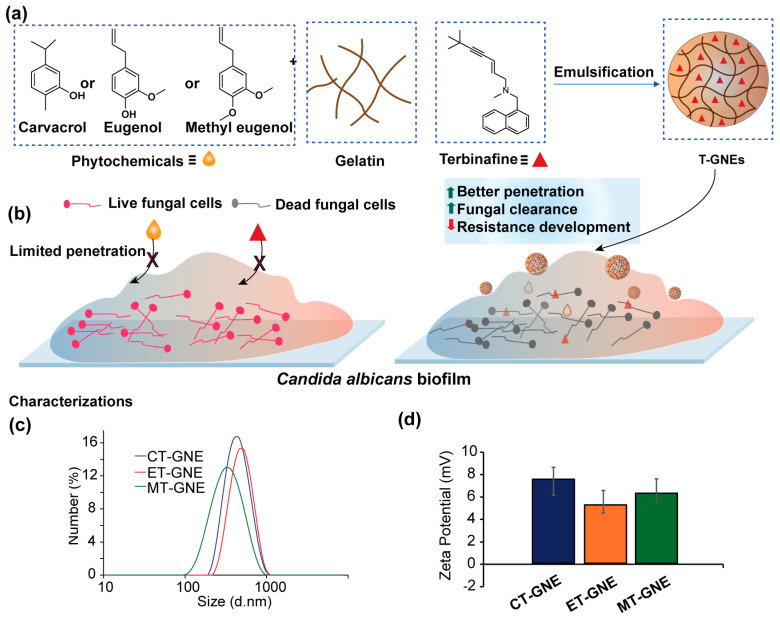
(**a**) Schematic representation of terbinafine-essential oil-loaded gelatin nanoemulsion (T-GNE) fabrication through emulsification. (**b**) Representation of efficient biofilm penetration of T-GNEs, compared to poor penetration of essential oil and terbinafine alone due to their hydrophobicity. Characterization of T-GNEs via (**c**) DLS and (**d**) zeta potential.

T-GNE was fabricated with different essential oils: carvacrol, eugenol, and methyl eugenol (Figure 1a) [26]. The oils are GRAS with known broad-spectrum antifungal activity against planktonic fungi [20,32]. The encapsulation of hydrophobic materials by the gelatin scaffold was anticipated to enhance solubility and provide better penetration into the EPS matrix of biofilm, as well as the targeting of fungal cells (Figure 1b) [33]. Gelatin was used to encapsulate the essential oil with terbinafine as a combination therapy. Oil/gelatin ratios (Section 2.1) were chosen to provide ~300 nm particles; lower oil ratios would be expected to provide smaller particles with a lower loading of antimicrobials, while higher oil ratios would provide less stable and larger particles. The hydrodynamic size of T-GNEs was measured through DLS, which demonstrated a diameter of ~300 nm for MT-GNE, ~350 nm for ET-GNE, and ~360 nm for CT-GNE (Figure 1c). The zeta potential charge characterization revealed a positive charge, ranging from +5 to +8 mV, attributed to the cationic nature of the gelatin polymer at a low pH (5.0) (Figure 1d).

### 3.2. Assessment of Minimal Inhibitory Concentrations (MICs)

We first assessed the MICs of terbinafine and GNEs, including carvacrol, eugenol, and methyl eugenol-loaded gelatin nanoemulsions (C-GNEs, E-GNEs, and M-GNEs), respectively, against *C. albicans* IDRL-7034. The MIC values for C-GNE 4%, E-GNE 8%, and M-GNE 16% are as illustrated in Table S1. Terbinafine alone showed that 32 µg/mL inhibited the growth of planktonic *C. albicans* (Table S1). The integration of terbinafine with C-GNEs, E-GNEs, and MT-GNEs showed a 4-fold decrement in the MIC of CT-GNE value and a 2-fold change in the MICs of ET-GNE and MT-GNE (Table S1).

### 3.3. Minimal Biofilm Inhibitory Concentration (MBIC) Analysis

**Figure 2:**
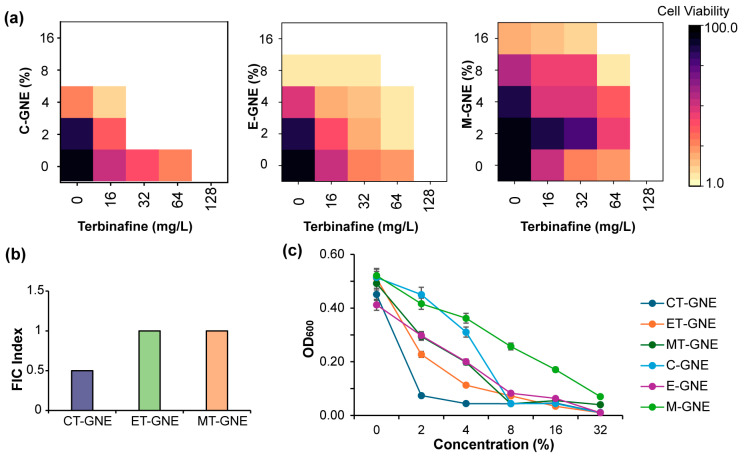
Checkerboard microdilution assay of essential oils combined with terbinafine into gelatin nanoemulsions (T-GNEs) against *C. albicans* IDRL-7034 based on minimal biofilm inhibitory concentration (MBIC). (**a**) A 2D checkerboard microdilution assay of carvacrol/eugenol/methyl eugenol-loaded gelatin nanoemulsions (C-GNEs) incorporation with terbinafine (16–128 mg/L). (**b**) Fractionation inhibitory concentration (FIC) indices correspond to synergy (≤0.5) for CT-GNE and exhibit additive effect (0.5 < FIC Index ≤ 1) for ET-GNE and MT-GNE. (**c**) Comparison between the growth curve of GNEs and T-GNEs based on MBIC values.

The MBICs of terbinafine and GNEs, including carvacrol, eugenol, and methyl eugenol-loaded gelatin nanoemulsions (C-GNEs, E-GNEs, and M-GNEs), were assessed against *C. albicans* IDRL-7034 (Figure 2a). The MBICs were 8% for C-GNE, 16% for E-GNE, and 32% for M-GNE, whereas the terbinafine MBIC was 128 µg/mL (Figure S1a). The incorporation of approximately 800 µg of terbinafine per mL of GNEs (100%), compared to GNEs alone, enhanced the MBICs; specifically, the MBIC values were as follows: 2% (with 32 µg/mL of terbinafine) for CT-GNE, 8% (with 64 µg/mL of terbinafine) for ET-GNE, and 16% (with 64 µg/mL of terbinafine) for MT-GNE (Figure 2c).

### 3.4. Evaluation of Fractional Inhibitory Concentration (FIC)

Combination therapies offer promise to treat fungal biofilms, reduce the selection of drug resistance, and improve the penetration of drugs into the biofilm matrix with minimum toxicity [34,35]. The synergy of two antimicrobial agents may potentially allow for reduced dosages [29]. The combination effect of carvacrol, eugenol, and methyl eugenol, with terbinafine incorporated into gelatin nanoemulsions, against *C. albicans* IDRL-7034 was assessed using a 2D checkerboard microdilution assay [36]. Different concentrations (1–16%) of C-GNE, E-GNE, and M-GNE were combined with varying concentrations of terbinafine (16–128 mg/L) against *C*. *albicans* and the FIC indices were calculated (Figure 2b). The checkerboard assay revealed a positive interaction between C-GNE and terbinafine, as confirmed by the enhanced MBIC activity, as compared to GNE (Figure 2a). FIC indices demonstrated that a synergistic interaction was found between C-GNE and terbinafine (FIC indices ≤ 0.5) (Figure 2a), while the combination of E-GNE and M-GNE with terbinafine showed additive effects (0.5 < FIC index ≤ 1) (Figure 2b).

### 3.5. Antibiofilm Activity Assessment of T-GNE

**Figure 3:**
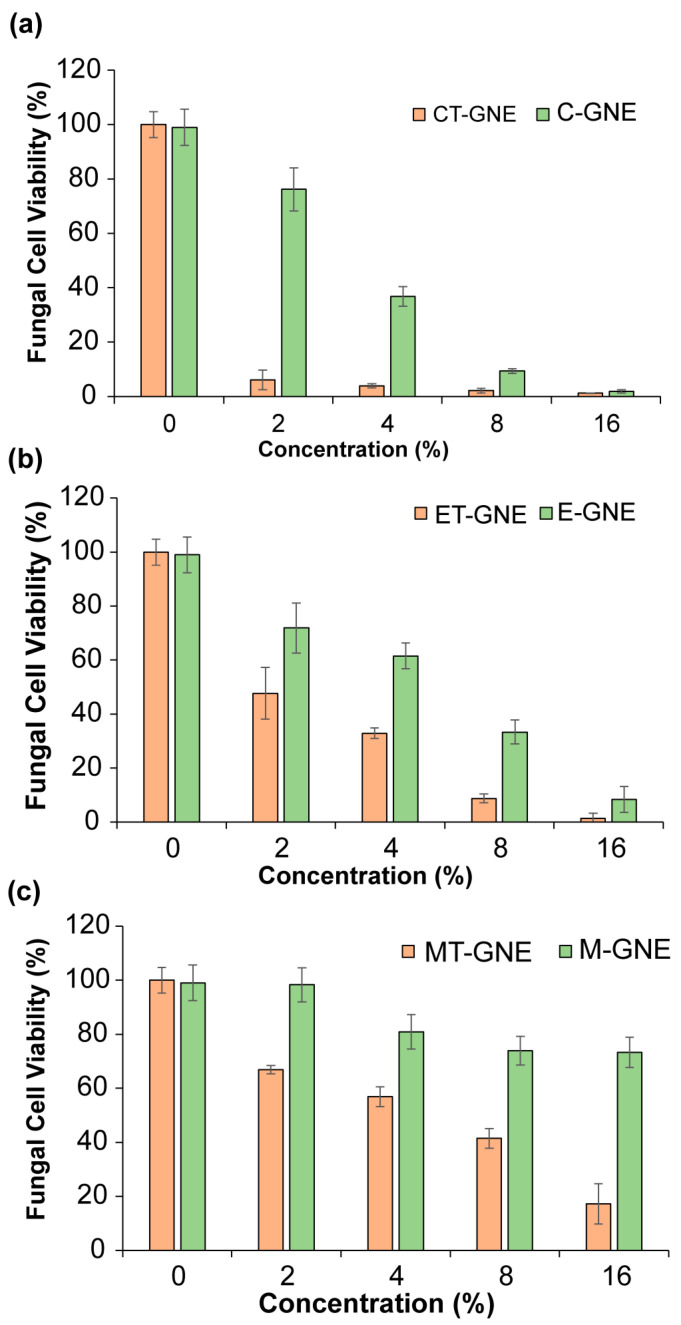
Comparison of the antibiofilm activity of terbinafine-essential oil-loaded gelatin nanoemulsions (T-GNEs) and essential oil-loaded gelatin nanoemulsions (GNEs) alone against 2-day-old *Candida albicans* IDRL-7034 biofilm showing effective antifungal activity, (**a**) antibiofilm efficacy of CT-GNE and C-GNE (**b**) ET-GNE and E-GNE, and (**c**) MT-GNE and M-GNE. Values are expressed as mean ± standard deviation of ≥3 replicates.

The antibiofilm efficacy was evaluated by treating a 2-day-old biofilm of *C. albicans* IDRL-7034 for 3 h. The fungal cell viability was assessed using the Alamar Blue assay. The incorporation of terbinafine into GNEs enhanced antibiofilm activity, as compared to GNEs alone. An approximately 4-fold increment in antibiofilm activity was observed with 2% CT-GNE, which killed 95% of fungal cells, compared to C-GNE alone. Additionally, a 2-fold enhancement in activity against biofilms was detected for ET-GNE and MT-GNE incorporated with terbinafine (Figure 3a–c). Significantly, terbinafine alone was ineffective even at high concentrations (512 mg/L), as shown in Figure S1b.

### 3.6. Biofilm Penetration Study

**Figure 4:**
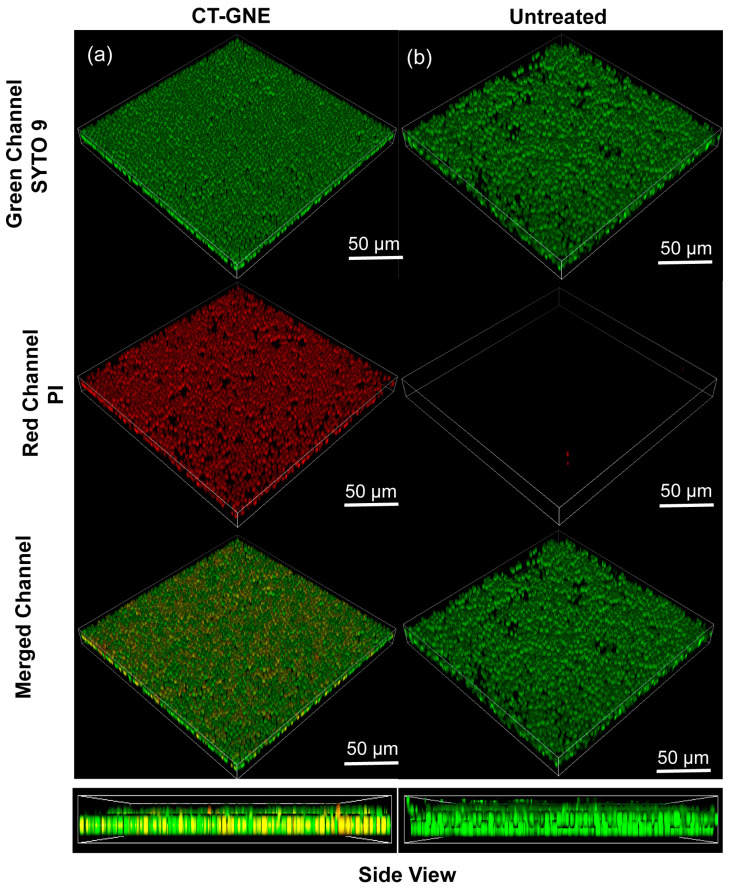
Confocal laser scanning microscopy (CLSM) images of 2-day-old *Candida albicans* IDRL-7034 biofilm stained by PI (Red) and SYTO 9 (Green) (**a**) treated with CT-GNE, showing complete co-distribution of fluorescence and hence, complete biofilm penetration, (**b**) untreated as representative 3D views.

Next, a biofilm penetration study of CT-GNE was performed and its impact on membrane integrity was investigated with a membrane-impermeable dye [37]. For this, live and dead fungal cell staining was used, including the SYTO-9 dye, which stains live and dead fungal cells, and PI, which stains cells with compromised membrane integrity, indicating cell death [35,37]. Two-day-old *C. albicans* biofilms were treated with an 8% CT-GNE for 2 h, followed by staining with SYTO-9 (green) and PI (red). The confocal micrograph revealed the co-localization of the green with the red fluorescence signal, indicating the penetration into the biofilm and the effect on fungal cell membrane integrity [38,39], in contrast to the untreated control (Figure 4).

### 3.7. Fibroblast Cells Viability with T-GNEs

**Figure 5:**
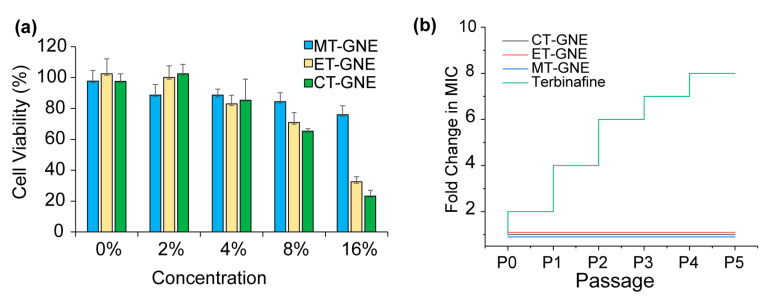
(**a**) Viability of 3T3/NIH fibroblast cells (ATCC CRL-1658) after 3 h exposure to T-GNEs. (**b**) Resistance development against T-GNEs and terbinafine, showing fold changes in MICs across successive passages. Significantly, no resistance development was observed with any of the GNE systems.

The cytotoxic effects of T-GNEs on NIH/3T3 human fibroblast cells (ATCC CRL-1658) were evaluated; fibroblasts play a crucial role in the wound-healing process and interact with *C. albicans* during wound infection [40,41]. The seeded cells were treated with concentrations of T-GNEs ranging from 2 to 16% for 3 h and the viability of fibroblast cells were evaluated through Alamar Blue assays. Higher concentrations of T-GNEs showed that the cytotoxic effect of mammalian cell viability reduced to 30–40%. However, effective concentrations of CT-GNE (2%), ET-GNE (8%), and MT-GNE (16%) against *C. albicans* biofilms exhibited minimal impact on mammalian cell viability, as shown in Figure 5a.

### 3.8. Resistance Response to Sub-Inhibitory Concentration

Next, the development of resistance in *C. albicans* was assessed against CT-GNE, ET-GNE, and MT-GNE, as well as terbinafine alone, since the prolonged or repeated antifungal treatment during recurrent infections can lead to fungal resistance against these drugs [42]. Resistance development was evaluated over five passages by treating *C. albicans* with sub-inhibitory concentrations [43] of CT-GNE, ET-GNE, MT-GNE or terbinafine. As shown in Figure 5b, the MIC of terbinafine increased with each passage, resulting in a 9-fold increment in MIC after five passages. In contrast, no resistance development was observed against any T-GNE formulations. These results show that combining essential oils with antifungal drugs within a gelatin polymeric scaffold system can mitigate the selection of resistance.

## 4. Discussion

Fungal biofilms can transform simple infections into life-threatening conditions [44]. Phytochemicals, such as essential oils, can be used as a strategy to address biofilm-associated infections. Essential oils have broad-spectrum antimicrobial actions and a low potential for resistance development [45,46]. The efficacy of numerous essential oils against fungal infections has been investigated, but their applicability is still infective against biofilm infections due to their poor stability and limited penetration into the complex microenvironment of biofilms [15,47].

Nanoemulsion systems offer a viable option to overcome the physical barrier of EPS and deliver hydrophobic drugs across the biofilm [45,48]. Gelatin, a natural and GRAS polymer, can act as the nanocarrier platform to deliver hydrophobic antimicrobials across the biofilm matrix efficiently [26]. The gelatin polymeric framework consists of a hydrophobic and hydrophilic matrix, which has been used to encapsulate the essential oil and enhance their solubility by delivering them inside the microenvironment of fungal biofilms [23]. The combination approach is a substantial platform to enhance antimicrobial activity with the benefit of minimal drug resistance through its multimode mechanism of action [28,49]. The antifungal potential of essential oils against *C. albicans* has been enhanced with the integration of antifungal drugs [50]; however, studies on testing the combinational effect of essential oils with antifungal drugs, especially terbinafine, against *C. albicans* biofilm are still limited. This study provides an insight into enhancing the antibiofilm efficacy of essential oils through the addition of antifungal drugs, effectively targeting the fungal biofilm.

In this study, a gelatin-based nanoemulsion system was fabricated by loading essential oils and terbinafine into gelatin. The scaffold was developed as a combination therapy for treating *C. albicans* biofilm infections. When combined with terbinafine, C-GNE showed a synergistic effect, while E-GNE and M-GNE showed additive effects. The results revealed that combining terbinafine with C-GNE showed a 4-fold increase in antifungal efficacy, achieving 95% biofilm eradication, compared to C-GNE alone (Figure 3). ET-GNE and MT-GNE caused a 90% reduction in fungal cell viability at 8% and 16% concentrations, respectively. These results showed a two-fold increment in the antibiofilm activity of ET-GNE and MT-GNE, as compared to E-GNE and M-GNE alone. Additionally, terbinafine alone did not eradicate the fungal biofilm, even at a concentration of 512 mg/L (Figure S1b). These results are in accordance with previous studies indicating the enhanced efficacy of essential oils and antifungal drugs loaded into a delivery system [51,52]

Further, CLSM showed that at 8%, CT-GNE reduced a 2-day *C. albicans* biofilm by disrupting the fungal cell membranes, as assessed by the live/dead staining assay. The presence of red fluorescence (PI) in treated biofilms, in contrast to the untreated control, indicates the membrane disruption and cell death of nearly all of the cells (Figure 4b). Thus, we hypothesize the mechanism of action for T-GNEs involved in disrupting fungal cell membranes [20].

The biocompatibility of T-GNEs on human 3T3 fibroblasts was assessed. The findings demonstrated that effective concentrations of CT-GNE (2%), ET-GNE (8%), and MT-GNE (16%) against *C. albicans* biofilms had minimal effects on mammalian cells, with more than 90% cell viability.

A resistance development study was performed to study the development of resistance in fungi in response to prolonged antifungal treatment [53]. *C. albicans* were exposed to sub-inhibitory concentrations of each CT-GNE, ET-GNE, and MT-GNE, along with terbinafine, in each passage. As shown in Figure 5b, the MIC of terbinafine increased, resulting in a 9-fold increment (512 mg/L). In contrast, there was no increment observed in the MICs of T-GNEs in up to five passages. Thus, the incorporation of terbinafine and essential oil in gelatin nanoemulsions provides an effective combination therapy against *C. albicans* biofilms without selecting resistance against this T-GNE system.

## 5. Conclusions

This work highlights the promising efficacy of T-GNE as a transformative approach for treating *C. albicans* biofilm-associated infections. Incorporating essential oils, especially carvacrol, with terbinafine in the CT-GNE formulation enhanced activity, demonstrating an ability to penetrate and reduce fungal cells, compared to other formulations. The synergistic effects of CT-GNEs overcome challenging barriers presented by biofilms while ensuring minimal toxicity to mammalian cells. Additionally, T-GNEs exhibited an advantage in preventing resistance development, a prevalent issue with conventional antifungal therapies. These promising results demonstrate that the CT-GNE is a potent candidate for addressing biofilm-associated and drug-resistant fungal infections with minimal cytotoxcity. Significantly, the use of GRAS components reduces the potential issues of clinical safety and environmental impact. Additionally, the nanoemulsion fabrication process is scalable, facilitating the broad use of the GNE systems. Further in vivo studies, along with pre-clinical research, are needed to validate the therapeutic efficacy of the combination therapy. These efforts will help to refine their application, opening the door to practical and effective solutions for combating biofilm-associated fungal infections in clinical use and other antifungal applications.

## Data Availability

The original contributions presented in this study are included in the article/Supplementary Material. Further inquiries can be directed to the corresponding author.

## Supplementary Materials

The following supporting information can be downloaded at: https://www.mdpi.com/article/10.3390/microorganisms13010127/s1. MBIC and the antibiofilm activity of terbinafine (Figure S1a,b), MICs of GNEs and terbinafine (Table S1) (PDF).

## References

[1] van Rhijn N., Arikan-Akdagli S., Beardsley J., Bongomin F., Chakrabarti A., Chen S.C., Chiller T., Lopes Colombo A., Govender N.P., Alastruey-Izquierdo A. (2024). Beyond Bacteria: The Growing Threat of Antifungal Resistance. Lancet.

[2] Lohse M.B., Gulati M., Johnson A.D., Nobile C.J. (2018). Development and Regulation of Single-and Multi-Species *Candida albicans* Biofilms. Nat. Rev. Microbiol..

[3] Benedict K., Jackson B.R., Chiller T., Beer K.D. (2019). Estimation of Direct Healthcare Costs of Fungal Diseases in the United States. Clin. Infect. Dis..

[4] Richardson J.P. (2022). *Candida albicans*: A Major Fungal Pathogen of Humans. Pathogens.

[5] Lee Y., Robbins N., Cowen L.E. (2023). Molecular Mechanisms Governing Antifungal Drug Resistance. NPJ Antimicrob. Resist..

[6] Talapko J., Juzbašić M., Matijević T., Pustijanac E., Bekić S., Kotris I., Škrlec I. (2021). *Candida albicans*—The Virulence Factors and Clinical Manifestations of Infection. J. Fungi.

[7] Whaley S.G., Berkow E.L., Rybak J.M., Nishimoto A.T., Barker K.S., Rogers P.D. (2017). Azole Antifungal Resistance in *Candida albicans* and Emerging Non-*albicans Candida* Species. Front. Microbiol..

[8] Lee Y., Puumala E., Robbins N., Cowen L.E. (2020). Antifungal Drug Resistance: Molecular Mechanisms in *Candida albicans* and Beyond. Chem. Rev..

[9] Rajendran R., Sherry L., Lappin D.F., Nile C.J., Smith K., Williams C., Munro C.A., Ramage G. (2014). Extracellular DNA Release Confers Heterogeneity in *Candida albicans* Biofilm Formation. BMC Microbiol..

[10] Rabin N., Zheng Y., Opoku-Temeng C., Du Y., Bonsu E., Sintim H.O. (2015). Biofilm Formation Mechanisms and Targets for Developing Antibiofilm Agents. Future Med. Chem..

[11] Chen H., Zhou X., Ren B., Cheng L. (2020). The Regulation of Hyphae Growth in *Candida albicans*. Virulence.

[12] D’agostino M., Tesse N., Frippiat J.P., Machouart M., Debourgogne A. (2019). Essential Oils and Their Natural Active Compounds Presenting Antifungal Properties. Molecules.

[13] Nazzaro F., Fratianni F., Coppola R., De Feo V. (2017). Essential Oils and Antifungal Activity. Pharmaceuticals.

[14] McClements D.J. (2020). Advances in Nanoparticle and Microparticle Delivery Systems for Increasing the Dispersibility, Stability, and Bioactivity of Phytochemicals. Biotechnol. Adv..

[15] Chang Y., McLandsborough L., McClements D.J. (2013). Physicochemical Properties and Antimicrobial Efficacy of Carvacrol Nanoemulsions Formed by Spontaneous Emulsification. J. Agric. Food Chem..

[16] Nayak R., Rai V.K., Pradhan D., Halder J., Rajwar T.K., Dash P., Das C., Mishra A., Mahanty R., Saha I. (2024). Exploring the Biofilm Inhibition Potential of a Novel Phytic Acid-Crosslinked Chitosan Nanoparticle: In vitro and in vivo Investigations. AAPS PharmSciTech..

[17] Brito G.S., Dutra R.P., Pereira A.L.F., Ferreira A.G.N., Neto M.S., Holanda C.A., Fidelis Q.C. (2024). Nanoemulsions of essential oils against multi-resistant microorganisms: An integrative review. Microb. Pathog..

[18] Padaraju A., Dwivedi F., Kumar G. (2023). Microemulsions, nanoemulsions and emulgels as carriers for antifungal antibiotics. Ther. Deliv..

[19] Silva A.C., Silvestre A.J., Vilela C., Freire C.S. (2021). Natural Polymers-Based Materials: A Contribution to a Greener Future. Molecules.

[20] Madkhali O.A. (2023). Drug Delivery of Gelatin Nanoparticles as a Biodegradable Polymer for the Treatment of Infectious Diseases: Perspectives and Challenges. Polymers.

[21] Uhlířová R., Langová D., Bendová A., Gross M., Skoumalová P., Márová I. (2023). Antimicrobial Activity of Gelatin Nanofibers Enriched by Essential Oils against *Cutibacterium acnes* and *Staphylococcus epidermidis*. Nanomaterials.

[22] Alsakhawy S.A., Baghdadi H.H., El-Shenawy M.A., Sabra S.A., El-Hosseiny L.S. (2022). Encapsulation of *Thymus vulgaris* Essential Oil in Caseinate/Gelatin Nanocomposite Hydrogel: In vitro Antibacterial Activity and In Vivo Wound Healing Potential. Int. J. Pharm..

[23] Nabawy A., Makabenta J.M., Li C.-H., Park J., Chattopadhyay A.N., Schmidt-Malan S., Gupta A., Patel R., Rotello V.M. (2020). Activity of Biodegradable Polymeric Nanosponges against Dual-Species Bacterial Biofilms. ACS Biomater. Sci. Eng..

[24] Oz Y., Nabawy A., Fedeli S., Gupta A., Huang R., Sanyal A., Rotello V.M. (2021). Biodegradable Poly (Lactic Acid) Stabilized Nanoemulsions for the Treatment of Multidrug-Resistant Bacterial Biofilms. ACS Appl. Mater. Interfaces.

[25] Nabawy A., Makabenta J.M., Schmidt-Malan S., Park J., Li C.-H., Huang R., Fedeli S., Chattopadhyay A.N., Patel R., Rotello V.M. (2022). Dual Antimicrobial-Loaded Biodegradable Nanoemulsions for Synergistic Treatment of Wound Biofilms. J. Controlled Release.

[26] Nabawy A., Makabenta J.M., Park J., Huang R., Nayar V., Patel R., Rotello V.M. (2024). Nature-Derived Gelatin-Based Antifungal Nanotherapeutics for Combatting *Candida albicans* Biofilms. Environ. Sci. Nano.

[27] Shakiba S., Astete C.E., Paudel S., Sabliov C.M., Rodrigues D.F., Louie S.M. (2020). Emerging Investigator Series: Polymeric Nanocarriers for Agricultural Applications: Synthesis, Characterization, and Environmental and Biological Interactions. Environ. Sci. Nano.

[28] Hassan H.A., Genaidy M.M., Kamel M.S., Abdelwahab S.F. (2020). Synergistic Antifungal Activity of Mixtures of Clove, Cumin and Caraway Essential Oils and Their Major Active Components. J. Herb. Med..

[29] Liu W., Li L.P., Zhang J.D., Li Q., Shen H., Chen S.M., He L.J., Yan L., Xu G.T., An M.M. (2014). Synergistic Antifungal Effect of Glabridin and Fluconazole. PLoS ONE.

[30] Gray D.A., Wenzel M. (2020). Multitarget Approaches against Multiresistant Superbugs. ACS Infect. Dis..

[31] Fan F., Liu Y., Liu Y., Lv R., Sun W., Ding W., Cai Y., Li W., Liu X., Qu W. (2022). *Candida albicans* Biofilms: Antifungal Resistance, Immune Evasion, and Emerging Therapeutic Strategies. Int. J. Antimicrob. Agents.

[32] Abd Rashed A., Rathi D.-N.G., Ahmad Nasir N.A.H., Abd Rahman A.Z. (2021). Antifungal Properties of Essential Oils and Their Compounds for Application in Skin Fungal Infections: Conventional and Nonconventional Approaches. Molecules.

[33] Chaleshtori A.S., Marzhoseyni Z., Saeedi N., Bahadori R.A., Mollazadeh S., Pourghadamyari H., Sajadimoghadam E., Abbaszadeh-Goudarzi K., Hasan-Abad A.M., Chaleshtori R.S. (2024). Gelatin-Based Nanoparticles and Antibiotics: A New Therapeutic Approach for Osteomyelitis?. Front. Mol. Biosci..

[34] Spitzer M., Robbins N., Wright G.D. (2017). Combinatorial Strategies for Combating Invasive Fungal Infections. Virulence.

[35] Aghaei Gharehbolagh S., Izadi A., Talebi M., Sadeghi F., Zarrinnia A., Zarei F., Darmiani K., Borman A.M., Mahmoudi S. (2021). New Weapons to Fight a New Enemy: A Systematic Review of Drug Combinations against the Drug-Resistant Fungus *Candida auris*. Mycoses.

[36] Bellio P., Fagnani L., Nazzicone L., Celenza G. (2021). New and Simplified Method for Drug Combination Studies by Checkerboard Assay. MethodsX.

[37] Stiefel P., Schmidt-Emrich S., Maniura-Weber K., Ren Q. (2015). Critical Aspects of Using Bacterial Cell Viability Assays with the Fluorophores Syto9 and Propidium Iodide. BMC Microbiol..

[38] Feldman M., Sionov R.V., Mechoulam R., Steinberg D. (2021). Anti-Biofilm Activity of Cannabidiol against *Candida albicans*. Microorganisms.

[39] Wang T., Shi G., Shao J., Wu D., Yan Y., Zhang M., Cui Y., Wang C. (2015). In vitro antifungal activity of baicalin against *Candida albicans* biofilms via apoptotic induction. Microb. Pathog..

[40] Li B., Wang J.H.-C. (2011). Fibroblasts and Myofibroblasts in Wound Healing: Force Generation and Measurement. J. Tissue Viability.

[41] von Müller C., Bulman F., Wagner L., Rosenberger D., Marolda A., Kurzai O., Eißmann P., Jacobsen I.D., Perner B., Hemmerich P. (2020). Active Neutrophil Responses Counteract *Candida albicans* Burn Wound Infection of Ex Vivo Human Skin Explants. Sci. Rep..

[42] Barrett T.C., Mok W.W.K., Murawski A.M., Brynildsen M.P. (2019). Enhanced Antibiotic Resistance Development from Fluoroquinolone Persisters after a Single Exposure to Antibiotic. Nat. Commun..

[43] Andersson D.I., Hughes D. (2014). Microbiological Effects of Sublethal Levels of Antibiotics. Nat. Rev. Microbiol..

[44] Ge Y., Wang Q. (2023). Current Research on Fungi in Chronic Wounds. Front. Mol. Biosci..

[45] Li C.H., Chen X., Landis R.F., Geng Y., Makabenta J.M., Lemnios W., Gupta A., Rotello V.M. (2019). Phytochemical-Based Nanocomposites for the Treatment of Bacterial Biofilms. ACS Infect. Dis..

[46] Allagui M.B., Moumni M., Romanazzi G. (2023). Antifungal Activity of Thirty Essential Oils to Control Pathogenic Fungi of Postharvest Decay. Antibiotics.

[47] Ahmad R., Srivastava S., Ghosh S., Khare S.K. (2021). Phytochemical Delivery Through Nanocarriers: A Review. Colloids Surf. B Biointerfaces.

[48] Garcia C.R., Malik M.H., Biswas S., Tam V.H., Rumbaugh K.P., Li W., Liu X. (2022). Nanoemulsion Delivery Systems for Enhanced Efficacy of Antimicrobials and Essential Oils. Biomater. Sci..

[49] Dhanda G., Acharya Y., Haldar J. (2023). Antibiotic adjuvants: A versatile approach to combat antibiotic resistance. ACS Omega.

[50] Blanc A.R., Sortino M.A., Butassi E., Svetaz L.A. (2023). Synergistic effects of *Thymus vulgaris* Essential Oil in Combination with Antifungal Agents and Inhibition of Virulence Factors of *Candida albicans*. Phytomed. Plus.

[51] Silva R.A.d., Silva N.B.S., Martins C.H.G., Pires R.H., Röder D.V.D.d.B., Pedroso R.d.S. (2022). Combining Essential Oils with Each Other and with Clotrimazole Prevents the Formation of *Candida* Biofilms and Eradicates Mature Biofilms. Pharmaceutics.

[52] Jafri H., Ahmad I. (2020). Thymus vulgaris essential oil and thymol inhibit biofilms and interact sinergistically with antifungal drugs against drug resistant strains of *Candida albicans* and *Candida Tropicalis*. J. Mycol. Med..

[53] Pereira R., Dos Santos Fontenelle R.O., de Brito E.H.S., de Morais S.M. (2021). Biofilm of *Candida albicans*: Formation, Regulation and Resistance. J. Appl. Microbiol..

