# Supplementary material for: Gelatin Nanoemulsion-Based Co-Delivery of Terbinafine and Essential Oils for Treatment of Candida albicans Biofilms
Source: Microorganisms. 2025 Jan 9;13(1):127. doi: 10.3390/microorganisms13010127 (PMC11767362; doi:10.3390/microorganisms13010127)
Supplement: Supplementary file 1 [file microorganisms-13-00127-s001.zip › microorganisms-3410271-supplementary.pdf]

Gelatin Nanoemulsion-Based Co-Delivery of Terbinafine and Essential Oils for Treatment of *Candida albicans* Biofilms

Muhammad Aamir Hassan [1], Sadaf Noor [1,2], Jungmi Park [1], AhmedNabawy [1], Maitri Dedhiya [1], Robin Patel [3] and Vincent M. Rotello [1,*]

[1] Department of Chemistry, University of Massachusetts Amherst, 710 North Pleasant Street, Amherst, MA 01003, USA; muhammadaami@umass.edu (M.A.H.); snoor@umass.edu (S.N.); jungmipark@umass.edu (J.P.); anabawy@umass.edu (A.N.); mdedhiya@umass.edu (M.D.)
[2] Institute of Molecular Biology and Biotechnology, Bahauddin Zakariya University, Multan 60800, Pakistan
[3] Division of Clinical Microbiology, Department of Laboratory Medicine and Pathology, Mayo Clinic, 200 First Street SW, Rochester, MN 55905, USA; patel.robin@mayo.edu
* Correspondence: rotello@chem.umass.edu

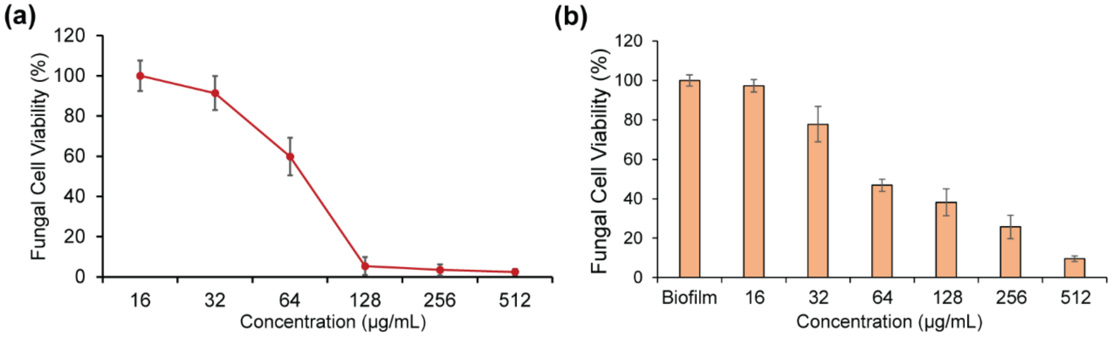

Figure S1: Antifungal activity of terbinafine. (a) Minimal biofilm inhibitory concentration of terbinafine. (b) Antibiofilm activity against two-day-old *Candida albicans* IDRL-7034 biofilm. Values are expressed as mean ± standard deviation of ≥3 replicates.

Table S1: Minimal inhibitory concentrations (MICs) of GNEs and terbinafine against *Candida albicans* IDRL-7034.

GNEs	MICs
C-GNE	4%
E-GNE	8%
M-GNE	16%
CT-GNE	1%
ET-GNE	4%
MT-GNE	8%
Terbinafine	32µg/mL

C: carvacrol; E: eugenol, M: Methyl Eugenol; T: Terbinafine
